# Complex and divergent histories gave rise to genome‐wide divergence patterns amongst European whitefish (*Coregonus lavaretus*)

**DOI:** 10.1111/jeb.13948

**Published:** 2021-10-26

**Authors:** Marco Crotti, Colin W. Bean, Andy R. D. Gowans, Ian J. Winfield, Magdalena Butowska, Josef Wanzenböck, Galina Bondarencko, Kim Præbel, Colin E. Adams, Kathryn R. Elmer

**Affiliations:** ^1^ Institute of Biodiversity, Animal Health & Comparative Medicine College of Medical, Veterinary & Life Sciences University of Glasgow Glasgow UK; ^2^ Scottish Centre for Ecology and the Natural Environment University of Glasgow Glasgow UK; ^3^ NatureScot Clydebank UK; ^4^ Environment Agency Penrith UK; ^5^ Lake Ecosystems Group UK Centre for Ecology & Hydrology Lancaster Environment Centre Bailrigg UK; ^6^ Research Institute for Limnology Mondsee, University of Innsbruck Mondsee Austria; ^7^ Vavilov Institute of General Genetics Ulitsa Gubkina Moscow Russia; ^8^ Norwegian College of Fishery Science UiT The Arctic University of Norway Tromsø Norway

**Keywords:** biogeography, population genomics, postglacial history, salmonid

## Abstract

Pleistocene glaciations dramatically affected species distribution in regions that were impacted by ice cover and subsequent postglacial range expansion impacted contemporary biodiversity in complex ways. The European whitefish, *Coregonus lavaretus*, is a widely distributed salmonid fish species on mainland Europe, but in Britain it has only seven native populations, all of which are found on the western extremes of the island. The origins and colonization routes of the species into Britain are unknown but likely contributed to contemporary genetic patterns and regional uniqueness. Here, we used up to 25,751 genome‐wide polymorphic loci to reconstruct the history and to discern the demographic and evolutionary forces underpinning divergence between British populations. Overall, we found lower genetic diversity in Scottish populations but high differentiation (*F*_ST_ = 0.433–0.712) from the English/Welsh and other European populations. Differentiation was elevated genome‐wide rather than in particular genomic regions. Demographic modelling supported a postglacial colonization into western Scotland from northern refugia and a separate colonization route for the English/Welsh populations from southern refugia, with these two groups having been separated for more than *ca*. 50 Ky. We found cyto‐nuclear discordance at a European scale, with the Scottish populations clustering closely with Baltic population in the mtDNA analysis but not in the nuclear data, and with the Norwegian and Alpine populations displaying the same mtDNA haplotype but being distantly related in the nuclear tree. These findings suggest that neutral processes, primarily drift and regionally distinct pre‐glacial evolutionary histories, are important drivers of genomic divergence in British populations of European whitefish. This sheds new light on the establishment of the native British freshwater fauna after the last glacial maximum.

## Graphical abstract

Using a dataset of genome‐wide polymorphic loci and population genomics approaches, we reconstructed the evolutionary history and discerned the demographic and evolutionary forces underpinning divergence between British populations of European whitefish. We found a deep divergence between Scottish and English/Welsh populations, with the latter sharing a more recent common ancestor with populations from central Europe.

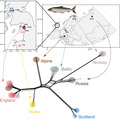

## 1 INTRODUCTION

Understanding the demographic and evolutionary forces shaping differentiation and speciation is a major undertaking in evolutionary biology (Seehausen et al., 2014). This is relevant not only for determining historical processes, but also to generate critical contemporary knowledge on how species are structured across their range, information that is important for conservation (Funk et al., 2012). Genomic differentiation can be rather uniform or localized in specific regions, known as islands of differentiation (Harr, 2006), which are usually considered as important genetic drivers of species divergence. Many factors will influence the distribution and magnitude of such regions (Quilodrán et al., 2020), and detailed knowledge of their patterns can improve the understanding of the evolutionary processes affecting species divergence. For example, divergent selection in populations experiencing gene flow, or secondary contact with heterogeneous gene flow following allopatric divergence, should result in few islands of high differentiation with modest and homogeneous background differentiation (Nosil et al., 2009; Ravinet et al., 2017). On the contrary, allopatric populations with limited or no gene flow may lack genomic islands of differentiation, resulting in a global and random pattern of genome‐wide differentiation due to drift (Mattingsdal et al., 2020; but see Cruickshank and Hahn, 2014). Furthermore, although gene flow can swamp genomic regions undergoing divergence, it has been shown that introgression of favourable alleles from divergent lineages or populations can facilitate local adaptation (e.g. Jacobs et al., 2019; Marques et al., 2019). The use of high throughput sequencing techniques enables us to investigate such genome‐wide patterns to better understand the evolutionary processes behind population and species divergence.

Because of the continuous expansion and contraction of the glaciers, Pleistocene glaciations are one of the most important events to have shaped the current distribution of flora and fauna in areas that were glaciated (Schmitt, 2007; Stewart & Lister, 2001). Many species were fragmented into lineages in separate refugia, which then experienced secondary contact at the end of the last ice age as they expanded their range into the areas previously covered by ice, leading to complex evolutionary histories (Bernatchez, 2004; Hewitt, 2004). The postglacial colonization of Britain is of particular interest from a biogeographic perspective because, with the exception of southern England, it was completely covered by ice during the Last Glacial Maximum (LGM) (Brooks et al., 2011; Clark et al., 2012).

Amongst temperate freshwater fish species, the genetic diversity and intra‐specific structuring of salmonids were notably impacted by Pleistocene glaciations (e.g. Bernatchez & Dodson, 1991; Brunner et al., 2001; Jacobs et al., 2020; Koskinen et al., 2002; Ostberg et al., 2009; Præbel et al., 2013; Rougemont & Bernatchez, 2018). The European whitefish, *Coregonus lavaretus*, is such an example, with widespread phylogeographic structuring and the repeated evolution of distinct ecological ecotypes resulting from the interplay between secondary contact of previously allopatric lineages emerging during glacial periods and polygenic selection (Rougeux et al., 2017; Rougeux, Gagnaire, & Bernatchez, 2019; Rougeux, Gagnaire, Præbel, et al., 2019). Furthermore, the same genomic regions responsible for the divergence of European whitefish are behind the parallel evolution of different ecotypes in the sister species lake whitefish *C. clupeaformis* (Rougeux et al., 2017; Rougeux, Gagnaire, Præbel, et al., 2019), suggesting the contribution of ancestral polymorphism in the repeated evolution of ecotypes in this genus more generally.

**FIGURE 1:**
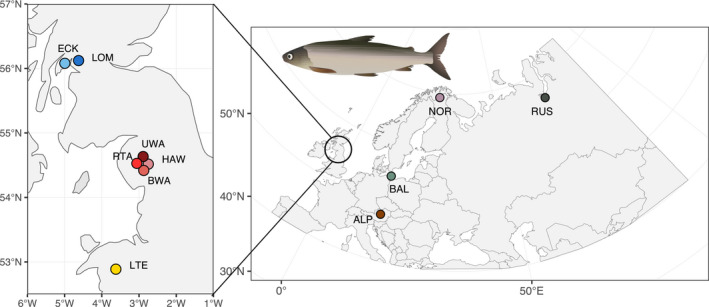
Map of the localities of the samples included in our studies. ALP, Koppentraun; BAL, Achterwasser; BWA, Brotherswater; ECK, Loch Eck; HAW, Haweswater; LOM, Loch Lomond; LTE, Llyn Tegid; NOR, Hávgajávri; RTA, Red Tarn; RUS, Ob; UWA, Ullswater. ECK and LOM are in Scotland, LTE in Wales, and RTA, HAW, BWA and UWA in England

The current understanding is that there are three major mitochondrial lineages of European whitefish that occur in Europe and survived in separate refugia: a northern European clade with contemporary populations ranging from northwest Russia to Denmark, a Siberian clade with populations ranging from the Arctic sea to southwest Norway and a southern European clade with populations ranging from the European Alps to Denmark (Jacobsen et al., 2012; Østbye et al., 2005). In Britain, the European whitefish is only native to seven lakes: Lochs Eck and Lomond in Scotland, Llyn Tegid in Wales, and Haweswater, Red Tarn, Brotherswater and Ullswater in England (Figure 1). Although the relationship between British and continental European populations is unknown, the genetic relationship between British populations has been investigated using allozymes (Beaumont et al., 1995; Ferguson, 1974), mitochondrial restriction fragment length polymorphism (RLFPs) (Hartley, 1995), and more recently with microsatellites (Crotti, Adams, Etheridge, et al., 2020). Although some contradictions exist between these different data types, they generally suggest a separation between populations in Scotland, Wales and England, with the Welsh population being more closely related to the English populations. However, these studies have lacked contextualization by continental Europe populations and were based on few genetic markers, and therefore it was not possible to infer the drivers of differentiation, the role of complex postglacial demography, and whether major genomic structural differences are involved. The European whitefish in the UK is a priority species within the UK Biodiversity Action Plan, and it is protected under Schedule 5 of the Wildlife and Countryside Act 1981 due to its rarity and vulnerability to pressures such as invasive species, water quality and habitat degradation (Maitland & Lyle, 2013; Winfield et al., 2013). Therefore, a better understanding of the relationship between the British populations, the drivers of the population structuring between countries and the evolutionary drivers of distinctiveness is of high importance not only for evolutionary understanding but also for conservation management and policy.

In this study, a high‐density genome‐wide and mitochondrial dataset for samples from populations across the European whitefish range, including all seven native British populations, was used to reconstruct the evolutionary history of European whitefish in Britain. To do so, we contextualize the contemporary genetic diversity and population structure of British European whitefish with other continental European populations and reconstruct the evolutionary history and demography in a coalescent framework. To infer the role of particular genomic regions in divergence, we investigate genome‐wide differentiation and admixture amongst populations. These results shed new light on the establishment of the native British freshwater fauna after the last glacial maximum and highlight the value of genome‐wide data in reconstructing evolutionary histories for conservation planning.

## 2 METHODS

### 2.1 Genomic data generation

We obtained a total of 102 *C. lavaretus* samples from 11 populations from across Eurasia (Figure 1): 10 individuals from Koppentraun in the Alpine region in Austria (ALP), 10 individuals from Achterwasser in the Baltic region in Germany (BAL), 10 individuals from Lake Hávgajávri in Norway (NOR), 4 individuals from the River Ob in Russia (RUS), 10 individuals from Loch Lomond (LOM) and 10 from Loch Eck (ECK) in Scotland, 11 individuals from Llyn Tegid in Wales (LTE), and from England, 11 individuals from Red Tarn (RTA), 4 individuals from Haweswater (HAW), 11 individuals from Ullswater (UWA) and 11 individuals from Brotherswater (BWA).

DNA was extracted from fish fin clips using the NucleoSpin Tissue kit (Macherey‐Nagel) following the manufacturer's recommendations. The protocol used for the ddRADseq (Peterson et al., 2012) library preparation follows Crotti, Adams, and Elmer (2020), which is modified from Recknagel et al. (2015). Briefly, 1 μg of genomic DNA per sample was double digested using the rare cutting enzyme *PstI*‐HF (CATCAG recognition site) and the common cutting enzyme *MspI* (CCGG recognition site). Combinatorial barcoded Illumina adapters were then ligated to *PstI*‐HF and *MspI* overhangs. Samples were size selected using a PippinPrep (Sage Science) at a tight target range of 150–300 bp fragments. To enrich for the selected loci, we performed PCR amplification cycles with the following settings: 30 s at 98°C, 9X (10 s 98°C, 30 s 65°C, 30 s 72°C), 5 min 72°C. After PCR purification, the library was run on a 1.25% agarose gel stained with SYBR Safe (Life Technologies) to remove any adapter dimers and/or fragments outside the selected size range. DNA was excised manually, cleaned and quantified using the Qubit Fluorometer with the dsDNA BR Assay (Life Technologies) to ensure the final library concentration of >1 ng/μl. The library was composed of 102 samples plus three technical replicates and was sequenced on an Illumina NextSeq500 with 75‐bp paired‐end reads at Glasgow Polyomics.

### 2.2 Data processing

First, raw reads were demultiplexed with *process_radtags* in *Stacks* v.2.4.1 (Catchen et al., 2013; Rochette et al., 2019) and both forward and reverse reads were retained, for a paired‐end approach. Reads with quality <20 were removed using *Trimmomatic* (Bolger et al., 2014). Reads were then mapped to a chromosome‐level assembly (GCA_902810595.1) of the European whitefish (De‐Kayne et al., 2020) using *bwa mem* v.0.7.17 (Li & Durbin, 2009) with default settings and retained if mapping quality was >20 with *samtools* v.1.7 (Li et al., 2009). A total of 10 individuals from eight populations did not pass QC due to low coverage and were excluded from the dataset. We assembled loci using *Stacks* v.2.4.1 and the *ref_map.pl* script. The raw dataset was further filtered depending on the type of analysis performed.

### 2.3 Data generation, genetic diversity and population structure

The *populations* programme in *Stacks* was used to generate a vcf file with the following settings: ‐*p* 9 (minimum number of populations a locus must be present in to be retained), ‐*r* 0.8 (minimum proportion of samples in a population required to have a locus), ‐‐*max_obs_het* 0.6 (maximum observed heterozygosity for a nucleotide at a locus), ‐‐*min_maf* 0.05 (minor allele frequency across populations required for a SNP to be included in the dataset) and ‐‐*write_single_snp* (retain one SNP per locus). This file was then filtered further with *vcftools* v.0.1.15 (Danecek et al., 2011), retaining SNPs that fulfilled the following criteria: a minimum sequencing depth of 5 reads per individual, a minimum mean sequencing depth of 10 reads across individuals, a maximum sequencing depth per individual of 30, a maximum mean sequencing depth across individuals of 30 (to remove possible repetitive reads), a minor allele frequency (MAF) of 0.05 and a 15% missing data threshold. We then used the script *filter_hwe_by_pop.pl* (available at https://github.com/jpuritz/dDocent/blob/master/scripts/filter_hwe_by_pop.pl) to filter sites that were out of HWE within populations, although no such sites were detected. Finally, we excluded a sample from the Baltic region which had more than 90% missing data after filtering. The Russian samples had between 68% and 87% missing data but were retained in the dataset only for the population structure and phylogenetic analyses. Through this process, we identified 25,751 RAD loci that were retained for the analysis (i.e. whitelist loci). Summary statistics of genetic diversity (expected heterozygosity [H_E_], observed heterozygosity [H_O_] and nucleotide diversity [π]) were calculated by the *population* module of *Stacks*. Rarefied allelic richness was calculated in *hierfstat* v. 0.04 (Goudet, 2005), set to a minimum of three individuals (the size of the HAW population). For all these analyses, we included up to 17 SNPs from the retained loci for a total of 41,929 SNPs, as there is no need to account for linkage disequilibrium.

Multiple approaches were used to assess population structure. For most of these analyses, we used the retained loci but only used one SNP from each (to reduce the effect of linkage), with a total of 91 samples, 25,751 SNPs, and an average of 12.4 (± 5.5) SNPs per 1 Mb; this is referred as the ‘pop‐gen dataset’. First, to evaluate population structure at the individual level, we ran a principal component analysis (PCA) with *SNPRelate* v. 1.16 (Zheng et al., 2012) in the R statistical environment (R Core Team, 2019). To investigate whether differentiation along principal components was driven by specific genomic regions, we used the function *snpgdsPCACorr* in *SNPRelate*, which calculates the correlation between a SNP genotype and the eigenvector of each principal component (Zheng et al., 2012). Second, we employed a maximum‐likelihood approach for population assignment with *Admixture* v.1.3.0 (Alexander et al., 2009). We ran analyses with a 20‐fold cross‐validation (CV), and tested *K* values ranging 2–11, the optimal value was assigned to the one with the lowest CV‐error. Third, to complement the model‐based *Admixture* with a nonparametric approach (Linck & Battey, 2019), we used the R package *adegenet* v.2.1.1 (Jombart, 2008) to run a Discriminant Analysis of Principal Components (DAPC) (Jombart et al., 2010), which uses *k*‐means clustering and the Bayesian information criterion to identify the most likely number of genetic clusters in the dataset. The *xvalDAPC* function was used to determine the number of PCs to be retained by the DAPC analysis. Fourth, between population Weir and Cockerham *F*_ST_ (Weir & Cockerham, 1984) values were calculated in *GenoDive* (Meirmans & Van Tienderenn, 2004) and significance was assessed with 10,000 permutations, pairwise *Dxy* distances were calculated with *populations* in *Stacks*, and both were visualized as heatmap matrices. Finally, to infer population structure via shared ancestry, we used the software *fineRADStructure* (Malinsky et al., 2018), which uses haplotype‐based statistics, and is a modified version of the *chromopainter*/*fineSTRUCTURE* package (Lawson et al., 2012) but designed specifically for RADseq‐type data. For this, we generated a new dataset specific for *fineRADStructure* with *populations*, excluding the Russian individuals due to high missing data (Malinsky et al., 2018). Starting from all retained loci, we retained all SNPs per locus and filtered loci with the following settings: ‐*p* 9, ‐*r* 0.9, ‐‐*max_obs_het* 0.6 and ‐‐*min_maf* 0.05. The *fineRADStructure* dataset had a total of 24,060 loci and 87 individuals. The analysis was conducted with a burn‐in of 300,000 iterations, followed by 300,000 MCMC iterations and tree building with default parameters. The results were visualized with the scripts *fineradstructureplot*.*r* and *finestructurelibrary*.*r* (available at http://cichlid.gurdon.cam.ac.uk/fineRADstructure.html).

We used *TREEMIX* v.1.13 (Pickrell & Pritchard, 2012) to explore and visualize admixture events across populations using genome‐wide allele frequency from the pop‐gen dataset. This programme allows modelling migration events by adding discrete mixture events to a bifurcating population tree. Variable numbers of migration edges *m*, ranging 0 to 11, were tested. To account for linkage disequilibrium, we run the analysis in blocks of 100 SNPs. Because of the lack of outgroup in our dataset, we did not specify a root for the tree.

To understand whether genetic differentiation between the Scottish and other populations was evenly distributed or localized in specific genomic regions, we calculated differentiation across the genome using a window‐based approach in *vcftools* with the pop‐gen dataset, using a window size of 1 Mb. We compared window‐based *F*_ST_ between the two Scottish and the remaining populations, and between the Baltic and the remaining populations, given its similar level of differentiation from all populations (see Results). Windows with fewer than five SNPs were excluded as they could be unreliable (Lehnert et al., 2020). Furthermore, we looked for regions associated with high divergence and considered as outliers any genomic windows with *F*_ST_ values ≥ 3 standard deviations from the mean (SD) (Lehnert et al., 2020). For this last analysis, as the aim was to determine outliers across whitefish lineages, we used only one representative population from Scotland (Loch Lomond) and one representative population from England (Red Tarn).

### 2.4 Phylogenetic analysis

For phylogenetic analyses, the pop‐gen dataset was converted to phylip format using the script *vcf2phylip.py* (Ortiz, 2019). Phylogenetic reconstruction was conducted under a maximum‐likelihood framework in *RAxML*‐*NG* (Kozlov et al., 2019), using a generalized time‐reversible accounting for among‐site rate variation (GTR+G). Branch support was estimated using 100 bootstrap replicates. The analysis was run on the CIPRES server (https://www.phylo.org/portal2/home.action). In addition, we also investigated the relationship of European whitefish populations using unrooted phylogenetic networks as implemented in *SplitsTree* v.4.14.6 (Huson & Bryant, 2006) using the same dataset.

### 2.5 Inference of evolutionary history

To distinguish between alternative evolutionary scenarios regarding the colonization of Britain, we used coalescence simulations implemented in *fastsimcoal2* v.2.6 (Excoffier et al., 2013). Six populations were used for this analysis: Norway, Alpine, Baltic, Lomond in Scotland, Llyn Tegid in Wales and Red Tarn in England. Only one population each from Scotland and England were used because the population structure analyses had revealed very close relationship between populations within these two regions. We generated a dataset using the filtering criteria as the pop‐gen dataset, the only difference being that no MAF filter was applied to retain informative low‐frequency sites (Jacobs et al., 2020). The script *sampleKgenotypesPerPop.py* (available at https://github.com/marqueda/SFS‐scripts) was then used to down‐sample each population to seven individuals, to avoid biases due to different sample sizes, and no missing data were allowed. The filtering resulted in a dataset of 42 individuals and 20,510 SNPs. The minor multidimensional site frequency spectra (MSFS) were generated with the script *easySFS.py* (available at https://github.com/isaacovercast/easySFS). To determine absolute values for divergence times and other inferred parameters, we corrected the number of monomorphic sites in the SFS (following Jacobs et al., 2018; Kautt et al., 2016). We used a hierarchical approach (Marques et al., 2019) by firstly optimizing a three populations model (3‐Pop) that included the Alpine, Baltic and Norwegian populations from the two major European whitefish lineages and their contact zone (Østbye et al., 2005). The likelihood of each model was maximized from 100 random starting parameters, 40 iterations and 100,000 coalescent simulations. Following previous work, an estimated mutation rate of 1x10^−8^ was used, as no accurate mutation rate for salmonids is available (Rougeux et al., 2017). Once the best 3‐Pop model was found, we created four population (4‐Pop), five population (5‐Pop) and six population (6‐Pop) models by sequentially adding the remaining populations. The final model was a 6‐Pop model including all populations. As the size of the MSFS increases exponentially when the number of populations is >2 and sample size is large, most of the entries in the SFS will be zero or very low values and be difficult to estimate correctly (Excoffier et al., 2013). Therefore, to reduce the number of zero values, the MSFS for all the models except the 6‐Pop were projected down to eight chromosomes per population, whereas the MSFS for the 6‐Pop model was projected down to six chromosomes per population (Jacobs et al., 2020). We estimated confidence intervals for the parameters of the 6‐Pop model using parametric bootstrap: the point estimates with the highest likelihood from the best fitting model as identified by *fastsimcoal* were used to simulate 50 MSFS, for which we then run 15 parameter optimizations, starting from the best parameters inferred from the observed data, and the parameter from the run with the highest likelihood for each simulated MSFS was used to compute the confidence intervals. Parameter ranges for all the models are reported in Table S1.

### 2.6 Mitochondrial analysis

We sequenced mtDNA for a subset of the populations from this study. From the extracted DNA, the whole NADH dehydrogenase subunit 1 (ND1) mtDNA gene (975 bp) was PCR amplified using the primers ND1_F_B1NDF: 5′‐TAA GGT GGC AGA GCC CGGTA‐3′ and ND1_R_B1NDR: 5′‐TTG AAC CCC TAT TAG CCA CGC‐3′ (Schenekar et al., 2014), using the PCR conditions described in Schenekar et al., (2014). The PCR product was sent to *DNA Sequencing and Services* (MRC PPU) for Sanger sequencing in the forward direction for all samples but two, which were sequenced in the reverse direction. Overall, we successfully amplified the mtDNA gene ND1 from the Scotland (*n* = 17), Wales (*n* = 5), Baltic (*n* = 5), Alpine (*n* = 6) and Norway (*n* = 10) populations; sequences were not available from the English populations. Contigs were aligned to a reference sequence in *Geneious* v.7.1.9 using the highest sensitivity method. The reference sequence (GenBank JQ661447) came from an individual from Ringkøbing Fjord in Denmark belonging to the southern European mtDNA haplogroup [as defined in Jacobsen et al. (2012)]. In addition to the reference, we downloaded 12 more sequences, three from the Baltic region of Estonia (JQ661382‐JQ661384), two from Ringkøbing Fjord (JQ661446, JQ661447), two from Tange lake in Denmark (JQ661433, JQ661434), three from Achterwasser in the Baltic coast of Germany (JQ661394‐ JQ661396), described by Jacobsen et al. (2012) and two samples of lake whitefish *Coregonus clupeaformis* from Lake Ontario in North America (MH301057, MH301058) (Schroeter et al., 2019) as outgroups. After alignment in Geneious v 7.1.9, we constructed a Median Joining Network in PopART (Leigh & Bryant, 2015).

## 3 RESULTS

### 3.1 Genetic diversity, population structure and admixture

**FIGURE 2:**
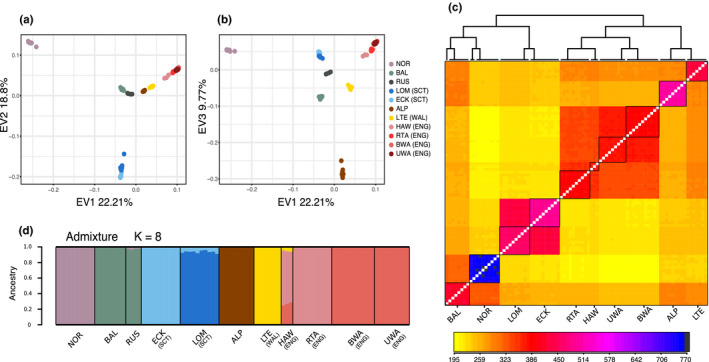
Analyses of population structure. (a) Principal component analysis showing PC1 and PC2, with each dot representing an individual and colour representing population. The first axis separates the Norwegian population, whereas the second axis separates the Scottish populations. (b) PCA showing PC1 and PC3. PC3 separates the Alpine population from all the others. (c) Co‐ancestry matrix from *fineRADStructure*, with populations delimited by black squares. Colours correspond to the level of low (yellow) and high (blue/black) co‐ancestry. The Scottish populations are very closely related to each other and are grouped with the Norwegian, Russian and Baltic population. (d) Ancestry coefficients from *Admixture* analysis. *K* = 8 was identified as the most likelihood number of clusters

To evaluate the extent to which European whitefish populations evolved independently or underwent significant admixture events, we analysed population structure, differentiation, co‐ancestry and admixture within Britain and at the European level. A PCA demonstrated that the Norwegian population was the most distinct from all other populations, separating along PC1, which explained 22.2% of the total variance (Figure 2a). The second principal component (18.8%) separated the two Scottish populations from all other populations, whereas the Alpine population separated on PC3 (9.77%, Figure 2b). The correlation analysis between SNP genotype and PC eigenvectors revealed a genome‐wide effect on the variation explained by the principal components (Figure S1), rather than specific genomic regions, suggesting that divergence between the Scottish and the other populations on PC2 is influenced by genome‐wide patterns rather than few elevated or adaptive regions.

All populations were significantly differentiated from each other, although there were substantial differences across geographic scales. We found the Scottish populations to be highly differentiated from the other British populations, with *F*_ST_ ranging from 0.546 to 0.661 (Table 1, Figure S4). Amongst populations within England, all *F*_ST_ values were <0.2, which was slightly lower than differentiation between the two Scottish populations (*F*_ST_ = 0.229). At the European level, the Baltic population was the least differentiated from all populations (*F*_ST_ = 0.298–0.502), whereas the population from Norway was the most differentiated (*F*_ST_ = 0.457–0.712) (Table 1, Figure S4), consistent with the PCA. The pattern of absolute divergence between populations, *Dxy*, was in agreement with that of *F*_ST_ (Figure S4), again with the Scottish populations being highly differentiated from other British populations.

**TABLE 1:** Weir & Cockerham F_ST_ calculated in *GenoDive* with 10,000 bootstrap replicates

	NOR	RUS	BAL	LOM (SCT)	ECK (SCT)	ALP	LTE (WAL)	HAW (ENG)	RTA (ENG)	BWA (ENG)	UWA (ENG)
NOR	–										
RUS	0.568	–									
BAL	0.457	0.314	–								
LOM	0.663	0.615	0.433	–							
ECK	0.712	0.692	0.502	0.229	–						
ALP	0.655	0.590	0.366	0.607	0.670	–					
LTE	0.610	0.515	0.298	0.546	0.616	0.467	–				
HAW	0.626	0.537	0.295	0.578	0.661	0.498	0.314	–			
RTA	0.628	0.549	0.355	0.569	0.626	0.497	0.351	0.094	–		
BWA	0.654	0.585	0.396	0.601	0.656	0.532	0.396	0.175	0.199	–	
UWA	0.647	0.574	0.377	0.593	0.652	0.523	0.380	0.152	0.179	0.035	–

Note

All comparisons were significant. The codes SCT, WAL and ENG identifies the population in Scotland, Wales and England, respectively.

Abbreviations: ALP, Koppentraun; BAL, Achterwasser; BWA, Brotherswater; ECK, Loch Eck; HAW, Haweswater; LOM, Loch Lomond; LTE, Llyn Tegid; NOR, Hávgajávri; RTA, Red Tarn; RUS, Ob; UWA, Ullswater.

The four measures of within‐population genetic variation (A_R_, H_E_, H_O_ and π) followed the same pattern across all populations (Table 2). Overall, genetic diversity was the lowest in the Scottish populations (H_O_ = 0.095–0.135), followed by the Alpine and Norwegian populations (H_O_ = 0.140–0.149), and it was intermediate in the English and Welsh populations (H_O_ = 0.157–180), and highest in the Baltic population (H_O_ = 0.244).

**TABLE 2:** Lakes and rivers sampled, sample abbreviations (Code), location (Country), sample size in the ‘pop‐gen dataset’ (N), rarefied allelic richness (A_R_), expected (H_E_) and observed (H_O_) heterozygosity calculated using only variant sites, nucleotide diversity (π) calculated using both variant and invariant sites

Lake/river	Code	Country	N	A_R_	H_E_	H_O_	π
Hávgajávri	NOR	Norway	10	1.22	0.138	0.149	0.00196
Achterwasser	BAL	Germany	8	1.38	0.235	0.244	0.00338
Ob	RUS	Russia	4	1.16	0.094	0.102	0.00120
Eck	ECK	Scotland	10	1.14	0.088	0.095	0.00125
Lomond	LOM	Scotland	10	1.20	0.124	0.135	0.00177
Koppentraun	ALP	Austria	9	1.21	0.130	0.140	0.00185
Llyn Tegid	LTE	Wales	7	1.27	0.165	0.178	0.00240
Haweswater	HAW	England	3	1.27	0.152	0.180	0.00234
Red Tarn	RTA	England	10	1.26	0.167	0.175	0.00238
Brotherswater	BWA	England	11	1.23	0.150	0.157	0.00212
Ullswater	UWA	England	9	1.24	0.154	0.165	0.00220

Note

All measures were calculated using 41,929 SNPs in 25,751 loci.

The ancestry analyses showed that most populations were separated in well‐defined clusters (Figure 2c,d). Nevertheless, high levels of haplotype sharing were evident between the two Scottish populations, and between the four English populations as recovered by *fineRADStructure* (Figure 2c). This was paralleled to a certain extent in the DAPC (*K* = 7) (Figure S2) and *Admixture* (*K* = 8) analyses (Figure 2d), which clustered the Scottish populations in one group or in two groups with shared ancestry respectively, and the English populations in two groups formed by Haweswater and Red Tarn and by Brotherswater and Ullswater. Furthermore, the English population of Haweswater showed signals of low shared ancestry with the Welsh population and with the cluster formed by the English Brotherswater and Ullswater populations (Figure 2d). At the European level, the Scottish populations were clustered together with the Norwegian and Baltic populations by *fineRADStructure*, whereas the English and Welsh populations clustered with the Alpine population (Figure 2c). The closer relationship between Scottish and northern European populations was also supported by the *Admixture* analysis for *K* = 2 (Figure S3). However, at *K* = 3, the Scottish populations formed their own ancestry cluster, which was present at a smaller frequency in the Russian, Baltic, Alpine and Welsh populations only (Figure S3).

The analysis in *TREEMIX* recovered instances of gene flow amongst most populations (Figure S5). The increase in likelihood due to the addition of migration edges approximately plateaued at *m* = 4 and stabilized at *m* = 7 (Figure S5). We found consistent evidence of gene flow between Norwegian and the Baltic populations, between Welsh and English populations, and between English populations (Figure S5). When including 6 to 11 migration edges, there is also evidence of weak admixture from the base of the Scottish population branch and the English populations.

Visualization of *F*_ST_ across windows showed a roughly even distribution in differentiation across the genome between Scottish and the Baltic populations (Figure S6), whereas an excess of regions of higher differentiation was observed between the Scottish and the remaining populations, and an excess of regions of low differentiation was observed between the two Scottish populations (Figure S6). The Baltic population showed the highest differentiation with the Scottish populations and an excess of genomic regions of low differentiation with the Welsh and English populations (Figure S6). When looking for genomic window outliers, we did not find any window with *F*_ST_ > 3 SD (Figure S7), again suggesting that differentiation between Scottish and other populations is driven by genome‐wide mechanisms rather than particular genomic regions.

### 3.2 Phylogenetic analysis

**FIGURE 3:**
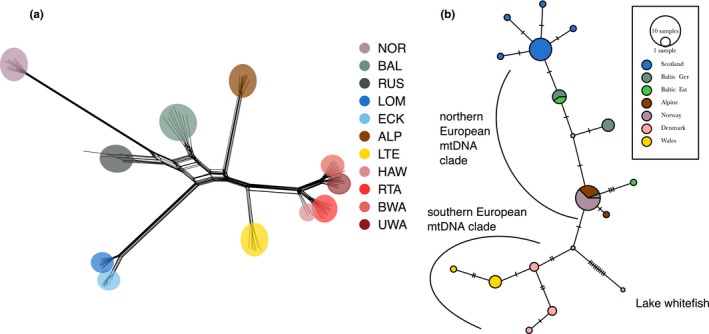
Results of phylogenetic and mitochondrial analyses. (a) Unrooted phylogenetic network calculated with SplitsTree using the genome‐wide SNPs data. (b) Median‐joining network calculated in PopART with ε = 0. Colour code same as in previous figures. Hash marks denote mutational steps, and the size of the circle is proportional to the haplotype frequency

The unrooted phylogenetic network based on genomic data showed populations to be well defined (Figure 3a), but highlighted the presence of unresolved lineage sorting between the Baltic, Russian and Norwegian populations. The topology of a phylogenetic analysis based on the same data agrees with the result of that phylogenetic network, with a high support for the monophyly of all populations except for Ullswater and Brotherswater in England (Figure S8a). With midpoint rooting applied, the Norwegian and Russian populations formed a monophyletic group sister to a clade containing all the other populations. The first population to split in this clade was the Baltic, followed by the two Scottish populations (Figure S8a). The four English populations were more closely related to the Welsh population, and these two lineages shared a more recent common ancestor with the Alpine population than with any other population (Figure S8a).

### 3.3 Mitochondrial analysis

Scottish populations possessed mitochondrial haplotypes that were predominantly in the northern European mtDNA clade (Figure 3b). The Welsh populations were placed in the southern European clade, being more similar to the Danish populations of Ringkøbing Fjord and Tange Lake (Jacobsen et al., 2012). The Scottish populations showed unique haplotypes, which were more closely related to Baltic ones (Figure 3b). Furthermore, the Norwegian and Alpine possessed the same haplotypes and were central in the haplotype network.

### 3.4 Evolutionary history

**FIGURE 4:**
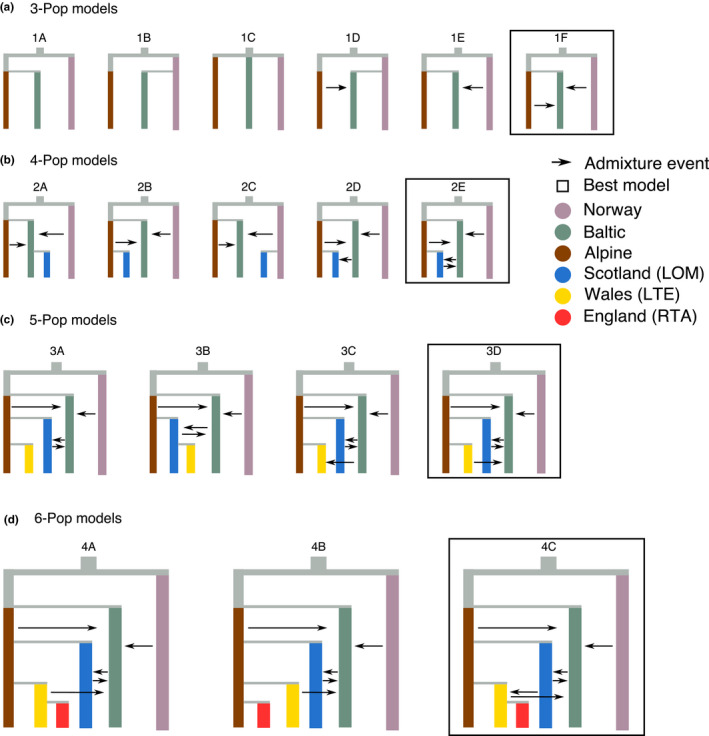
Hierarchical demographic modelling approach. (a) First, we established the relationship of three European whitefish populations, specifically the Norwegian, Baltic and Alpine. The data best supported a more recent split between the Baltic and Alpine populations, with the Baltic receiving gene flow from the other two populations. (b) Next, we tested the origin of the Scottish population, with the best supporting model suggesting a split between the Scottish and Alpine population, with genetic contribution from the Baltic population. (c) We then added the Welsh population, for which the modelling indicates a closer relationship with the Alpine population than with the Scottish one. (d) Finally, we tested the English population, which was found to be splitting from the Welsh population

Using the inference of phylogenetic relationships in the ML tree, we estimated the demographic history of European whitefish using a hierarchical approach. The demographic scenarios for all the models tested are found in Figure 4. The 3‐Pop model indicated the oldest divergence to be between the Norwegian and the group formed by the Alpine and Baltic population, with subsequent gene flow from the Alpine and Norwegian populations into the Baltic one (Figure 4a, Table S2). The 4‐Pop model showed the Scottish population of Loch Lomond splitting from the Alpine population, with additional gene flow with the Baltic population (Figure 4b, Table S2). In the 5‐Pop model, the Welsh population split from the Alpine population more recently than the Scottish population, and in the 6‐Pop model, the English population of Red Tarn shared a more recent common ancestor with the Welsh population (Figure 4c,d, Table S2). In both 5‐Pop and 6‐Pop models, the Baltic population received gene flow from the ancestor branch of the Welsh and English population (Figure 4c,d).

**FIGURE 5:**
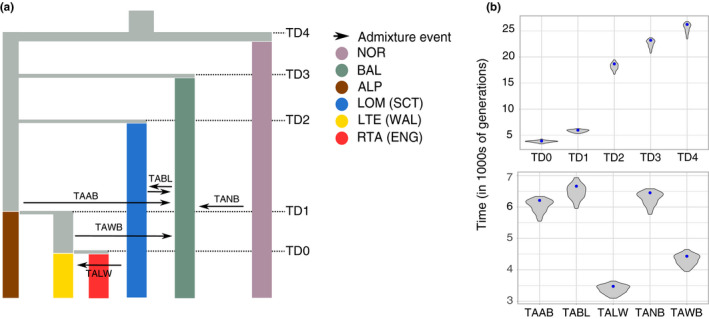
Demographic history of European whitefish. (a) The best fitting 6‐Pop model, with splitting and admixture events annotated. (b) Confidence intervals for the time of divergence and admixture events obtained by parametric bootstrapping, with maximum‐likelihood parameter estimates indicated by blue dots. Time unit is in generations before present

To infer coalescent times, confidence intervals were calculated for the 6‐Pop model using parametric bootstrapping (Table S3, Figure 5). We estimated the first divergence event, between the Norwegian population and the others, occurred 26,247 generations ago (CI: 23,451–26,774), the divergence between the Baltic population and the remaining occurred 23,205 (CI: 20,733–23,671), whereas the Scottish population diverged from the Alpine, Welsh and English populations 18,674 generations ago (CI: 16,685–19,435). On the contrary, the divergence between the Alpine and the Welsh/English populations, and between the Welsh and English populations, were more recent, the former having occurred 5965 generations ago (CI: 5313–6255) and the latter 3887 generations ago (CI: 3337–4259). Furthermore, our suggested inference that the Baltic population received gene flow from all other populations, with admixture proportion into the Baltic ranging from 0.15 from the Lomond population to 0.46 from the Norwegian population, between 6663 (CI: 5953–6934) and 4433 (CI: 3949–4649) generations ago. We also found evidence of low gene flow (admixture proportion = 0.15) from the Lomond to the Welsh population at 3473 (CI: 3093–3641) generations ago.

## 4 DISCUSSION

In our study to reconstruct the evolutionary and demographic history of European whitefish in Britain, analyses based on genome‐wide data support the role of historical genetic isolation due to demographic history and genetic drift as the main drivers of population differentiation. In fact, we found lower genetic diversity and higher differentiation in the Scottish populations compared with the other British and continental European populations, consistent with a scenario of low effective population size maintained over a long period of time in small glacial refugia (Westergaard et al., 2019). In addition, we found evidence of two separate routes for the colonization of Britain, as the Welsh and English populations shared a more recent common ancestor with an Alpine population than with the two Scottish populations. Finally, we inferred high levels of background genome‐wide differentiation between Scottish and remaining populations.

In agreement with the findings of previous studies based on mtDNA and microsatellites (Crotti, Adams, Etheridge, et al., 2020; Hartley, 1995), the Welsh population had the highest genetic variation within Britain and formed its own genetic cluster. The four English populations are very closely related, with high levels of within‐population genetic diversity and low between‐population genetic differentiation. Interestingly, although allozyme and microsatellite analyses suggested the English population of Red Tarn to be the most distantly related of the four (Beaumont et al., 1995; Crotti, Adams, Etheridge, et al., 2020), our SNPs analyses show a very close relationship between Red Tarn and Haweswater.

We found evidence of elevated genomic differentiation between the Scottish and the remaining populations across the entire genome but did not detect any genomic islands of differentiation. Although in an allopatric model of population divergence, the number of islands is expected to increase with time (Nosil et al., 2009), it has been shown that long periods of isolation and limited gene flow can mask the signals of genomic islands, as it will be swamped by elevated background genetic differentiation due to drift (Mattingsdal et al., 2020; Quilodrán et al., 2020; Wang et al., 2016). The high level of genetic differentiation between Scottish and all other populations could explain why we did not find specific genomic regions involved in divergence. Furthermore, the demographic modelling supports a deep separation between the Scottish and the remaining British populations. Assuming an average generation time of 3.5 years (Rougeux et al., 2017), our estimates indicate that the Scottish populations split from their common ancestor with the Alpine, Welsh and English populations ca. 65 Kya, and remained isolated from the British populations until ca. 12 Kya, when they experienced low gene flow with the ancestor of the Welsh and English populations. This extended period of isolation for this lineage is also evident from the long branch in the phylogenetic tree and network, and the strong separation on PC2. Nevertheless, our models did not explore potential additional factors that could impact the time of divergence estimation, such as the effect of bottlenecks followed by expansion and heterogeneous gene flow during divergence (Le Moan et al., 2016; Momigliano et al., 2021; Rougeux et al., 2017), so we acknowledge that some caution is needed when interpreting these results. We did not find evidence, in the form of elevated *F*_ST_ within specific chromosomes, of chromosomal rearrangements between Scottish and the other British and European populations. Despite an allopatric phase of ca. 15 to 60 Ky, an absence of large‐scale chromosomal rearrangements has been reported for lake whitefish species pairs (Dion‐Côté et al., 2015, 2017), suggesting karyotypic stability within *Coregonus*. However, substantial intrachromosomal polymorphism, particularly in regions associated with heterochromatin and repetitive DNA, was identified between sympatric species pairs of lake whitefish and vendace *Coregonus albula* (Dion‐Côté et al., 2017; Symonová et al., 2013), indicating that other mechanisms could be promoting differentiation in *Coregonus* populations.

A possible refugium for the Scottish lineage could have been located in the North Sea, as suggested for brown trout *Salmo trutta* (McKeown et al., 2010), bullhead *Cottus gobio* (Volckaert et al., 2002) and grayling *Thymallus thymallus* (Gum et al., 2005); alternatively, this lineage could have survived in the putative Lough Hibernia, in the present‐day Irish sea (Maitland, 1970). The Welsh and English populations split from the Alpine lineage much more recently, ca. 20 Kya, towards the end of the last glacial maximum (Clark et al., 2012). The Alpine lineage is thought to have had its refuge near the mouth of the river Rhine (Hudson et al., 2011) and colonized the Alpine lakes and the Jutland peninsula through the Rhine and Elbe river systems (Hansen et al., 2008; Østbye et al., 2005). At the time of the split, instead of moving towards central Europe, the ancestral stock that gave rise to the Welsh and English populations must have moved westwards towards Britain. The divergence between the English and Welsh populations at ca. 14 Kya agrees quite well with the retreat of the glaciers from Britain, as parts of the Lake District would have been already free of ice at that time (Brooks et al., 2011; Pinson et al., 2013). The subsequent Loch Lomond Stadial, or Younger Dryas, in Britain (12.9–11.7 Kya) (Bickerdike et al., 2018), a cold period which saw the re‐advancement of glaciers in Scotland, the English Lake District and Wales, could have promoted fragmentation of the whitefish populations in England.

Our results support a scenario in which European whitefish established in Britain in two different migratory waves, one formed by Welsh and English populations and one formed by the Scottish populations. Previous hypotheses suggested that *Coregonus* established in British lakes from an ancestral anadromous stock that was inhabiting Lough Hibernia (Maitland, 1970). Then as the glaciers started retreating, British lakes were colonized in different events; in fact, the Scottish lochs are low altitude, whereas the Welsh and English lakes are high altitude, suggesting that the European whitefish established in these water systems at different time points (Wheeler, 1977). This theory partially agrees with our finding of successive waves of colonization by European whitefish. However, we estimate a ca. 50k‐year‐old divergence and very limited gene flow between Scottish and remaining British populations, suggesting only a short phase of these lineages living in sympatry, or a longer phase of extensive reproductive isolation. This north/south genetic separation is unique amongst salmonids in Britain, which show diverse evolutionary histories. In Britain, the Arctic charr *Salvelinus alpinus* has a similar geographic distribution to whitefish, but to date, there is no evidence of high differentiation between British populations in mtDNA (Brunner et al., 2001; Wilson et al., 2004), and equivalent nuclear analyses have never been conducted. The grayling likely colonized Britain through the North Sea River from the central European lineage, but there is the possibility of a separate colonization for the northern England populations, as they contain both basal and extra haplotypes compared with southern English populations (Dawnay et al., 2011). British populations of brown trout and Atlantic salmon both descend from multiple genetic lineages, with evidence for subsequent hybridization between lineages (Finnegan et al., 2013; García‐Marín et al., 1999; McKeown et al., 2010). This suggests that species‐specific and geographically broad approaches are needed if we are to reconstruct the postglacial history of fishes in the British Isles.

The ancestors of the Norwegian population diverged from all other populations ca. 92 Ky ago, during a long interglacial stage know as GI 23.1 (Greenland Interstadial, Rasmussen et al., 2014) or MIS (Marine Isotope Stage) 5c (Oppo et al., 2001). During this period, temperatures increased and tree vegetation reached Scandinavia (Hall et al., 2002). The retreat of the glaciers could have allowed the ancestral stock of European whitefish to move westward. Through the early drainages of the ice lakes (Østbye et al., 2005), whitefish entered the Baltic sea around 81 Kya, corresponding to the interstadial GI 21.1 (Rasmussen et al., 2014) or MIS 5a (Oppo et al., 2001). The Baltic population in this study showed the highest genetic diversity. The high genetic diversity could be explained by repeated gene flow between the Baltic population and the other lineages, as suggested by the low differentiation, treemix results and the demographic modelling. These introgression events are also supported by mtDNA analysis (Østbye et al., 2005). In particular, the introgression between the ancestor of the Baltic and Alpine population could explain the presence of northern mtDNA haplotypes in the Alpine whitefish radiation (Hudson et al., 2011; Østbye et al., 2005). Overall, our results add support to the evidence of the Baltic sea as a recognized contact zone between divergent lineages of aquatic species (Johannesson et al., 2020).

Our analysis based on nuclear data does not find a clear distinction between a southern and a northern European whitefish clade, as suggested by mtDNA (Hudson et al., 2011; Jacobsen et al., 2012; Østbye et al., 2005). Instead, the demographic modelling finds a scenario in which three populations, Norwegian, Baltic and Scottish, split one after the other from the common ancestor of European whitefish within a 30 Ky period, from 92 to 65 Kya; then, we observe no split until 20 Ky, when the Alpine lineage splits from the Welsh and English stock. The Baltic population analysed here is from Achterwasser in northern Germany, and it was previously found to possess the northern mtDNA haplogroup (Jacobsen et al., 2012; Winkler et al., 2011). The fact that the demographic modelling shows the Baltic population splitting after the ancestor of the Norwegian population, rather than from it, adds support to the findings that the separation between southern and northern mtDNA haplogroups is not found in nuclear data. Furthermore, in our and previous analyses (Winkler et al., 2011), the northern European mtDNA haplotypes are prevalent in the Alpine population of Koppentraun, Austria. However, the nuclear data show a strong divergence between the Alpine and Norwegian populations, adding another line of evidence to the discrepancy between nuclear and mitochondrial DNA signal for European whitefish. Discordance between nuclear DNA and mtDNA is well documented in the literature (reviewed by Toews & Brelsford, 2012) and might be a common feature of species whose demographic history has been heavily influenced by glacial cycles (Dufresnes et al., 2020). Cyto‐nuclear discordance has also been recovered by previous studies on whitefish, in particular in the Alpine whitefish radiation, where nuclear data supported a monophyletic origin of all Alpine populations, whereas mtDNA showed the presence of both southern and northern mtDNA haplogroups (Hudson et al., 2011), and in the Danish and Baltic populations, where *F*_ST_ differentiation between Baltic (northern mtDNA haplogroup) and Danish (southern mtDNA haplogroup) was in some cases an order of magnitude higher for mtDNA than from microsatellite data (Hansen et al., 1999).

## 5 CONCLUSION

We provide molecular evidence of a deep divergence between, and a dual colonization of, Britain by Scottish and Welsh/English populations of European whitefish, which reflects a distinctive pre LGM pattern at the edge of the species distribution relevant to continental recolonization. Previous studies uncovered a similar structuring between the seven British populations (Beaumont et al., 1995; Crotti, Adams, Etheridge, et al., 2020; Hartley, 1995), but it is the first time it has been possible to estimate the time of divergence, highlighting the increased resolution NGS data can provide for conservation (Funk et al., 2012). Our findings support the delimitation of separate conservation units (CUs) (Funk et al., 2012) for each population and, in particular, highlight the uniqueness of the Scottish lineage which diverged and survived in isolation with limited contact for ca. 50K years. Although we did not detect genomic islands of differentiation between Scottish and remaining populations, future work could look at the adaptive differentiation and demographic history between populations within country, that is between the two Scottish populations and between the four English population, to better understand the adaptive and demographic process operating at the local level (Flanagan et al., 2018). Conservation measures have already been implemented to preserve the genetic stock of most British populations through the use of conservation translocations (Adams et al., 2014; Crotti et al., 2021; Maitland & Lyle, 2013; Thomas et al., 2013; Winfield et al., 2013), and we encourage national conservation bodies to look beyond the simple species‐based paradigm as they aim to protect the diversity of European whitefish at the most western edge of its range.

## CONFLICT OF INTEREST

The authors have no conflict of interest to declare.

## AUTHOR CONTRIBUTIONS

MC, CEA and KRE designed the research. MC, CWB, ARDG, IJW, JW, GB, KP and CEA provided biological samples. MC and MB conducted laboratory work with support from KRE. MC conducted the analyses. MC wrote the paper with contributions from KRE and CEA. All authors edited/approved the final manuscript.

### PEER REVIEW

The peer review history for this article is available at https://publons.com/publon/10.1111/jeb.13948.

## Supporting information

Supplementary Material

Table S1

## Data Availability

Scripts, data and the parameter data files for the demographic modelling in fastsimcoal are archived and made available in the University of Glasgow Enlighten Repository (http://dx.doi.org/10.5525/gla.researchdata.1059). Short read data are available at NCBI SRA (BioProject PRJNA648856) and mtDNA at GenBank (accessions MT862810–MT862852).
