# Supplementary material for: Complex and divergent histories gave rise to genome‐wide divergence patterns amongst European whitefish (Coregonus lavaretus)
Source: J Evol Biol. 2021 Oct 26;34(12):1954–69. doi: 10.1111/jeb.13948 (PMC9251650; doi:10.1111/jeb.13948)
Supplement: Supplementary file 1 — Supplementary Material [file JEB-34-1954-s002.docx]

**Supplemental Information for:**

# Complex and divergent histories gave rise to genome-wide divergence patterns amongst European whitefish (*Coregonus lavaretus*)

**Table of Contents:**

| **Figure S1.** Correlation between SNP genotype and the three principal components | **Page 2** |
| --- | --- |
| **Figure S2.** Discriminant Analysis of Principal Component (DAPC) | **Page 3** |
| **Figure S3.** Admixture results for K = 2 and K = 3. | **Page 4** |
| **Figure S4.** Dxy and F_ST_ heatmaps of all 11 populations. Dxy was calculated in Stacks, while F_ST_ in *GenoDive*. | **Page 5** |
| **Figure S5.** Results of the *TREEMIX* analysis. | **Page 6** |
| **Figure S6.** Density of F_ST_ across 1MB windows calculated in vcftools. | **Page 7** |
| **Figure S7.** Z-transformed Weir & Cockerham (1984) F_ST_ across 1MB windows | **Page 8** |
| **Figure S8.** Rooted and unrooted maximum likelihood tree | **Page 9** |
| **Table S1.** Fit of the demographic models to the observed minor site frequency spectra (MSFS). | **Page 10** |
| **Table S2.** Inferred parameters of the most likely model for the 6-POP dataset. | **Page 11** |

Figure S1. Correlation between SNP genotype and the three principal components from the PCA reported on Figure 2. Chromosomes are represented with different colours. Each cross represents a SNP.

Figure S2. Discriminant Analysis of Principal Component (DAPC). K = 7 was identified as best value. The analysis was based on ten retained principal components, as suggested by the cross-validation of DAPC.

Figure S3. Admixture results for K = 2 and K = 3.

Figure S4. Dxy and F_ST_ heatmaps of all 11 populations. Dxy was calculated in Stacks, while F_ST_ in *GenoDive*.

Figure S5. Results of the *TREEMIX* analysis. We included 0 to 11 migration edges, with the arrows indicating the direction of gene-flow, and colour indicating migration weight. We report the increase in ln(likelihood) for each additional migration edge, which starts stabilising at *m* = 7.

Figure S6. Density of F_ST_ across 1MB windows calculated in vcftools. The upper panel shows F_ST_ between the two Scottish populations (blue), between Scottish and the Baltic (green), the English (red), the Welsh (golden), the Alpine (brown), and the Norwegian (pink) populations. The lower panel shows differentiation between the Baltic and the Scottish (blue), the English (red), Welsh (golden), Alpine (brown), and Norwegian (pink) populations.

Figure S7. Z-transformed Weir & Cockerham (1984) F_ST_ across 1MB windows calculated in vcftools across each chromosome. A threshold of z-F_ST_ ≥ 3 was used to detect outlier windows.

Figure S8. Rooted and unrooted maximum likelihood tree on genomic data, obtained from RAxML on CIPRES. Colours and codes correspond to previous figures. Branch support was calculated using 100 bootstrap replicates.

Table S2 Fit of the demographic models to the observed minor site frequency spectra (MSFS). Best fitting models are marked with an asterisk (*). Shown are the differences in the Akaike Information Criterion (ΔAIC) between the best and remaining demographic models for each dataset.

| 3-Pop |  |  |  |  |
| --- | --- | --- | --- | --- |
| Model | ln lhood | N parameters | AIC | dAIC |
| A | -133082.24 | 6 | 266176.482 | 95.6347499 |
| B | -133536.77 | 6 | 267085.538 | 1004.69074 |
| C | -133197.94 | 5 | 266405.874 | 325.026131 |
| D | -133247.86 | 8 | 266511.728 | 430.880036 |
| E | -133062.57 | 8 | 266141.145 | 60.2973709 |
| F* | -133030.42 | 10 | 266080.848 | 0 |
|  |  |  |  |  |
| 4-Pop |  |  |  |  |
| Model | ln lhood | N parameters | AIC | dAIC |
| A | -151810.8 | 12 | 303645.597 | 215.577764 |
| B | -151736.34 | 12 | 303496.67 | 66.6511649 |
| C | -151983.07 | 12 | 303990.132 | 560.113571 |
| D | -151736.32 | 14 | 303500.638 | 70.6189287 |
| E* | -151700.01 | 15 | 303430.019 | 0 |
|  |  |  |  |  |
| 5-Pop |  |  |  |  |
| Model | ln lhood | N parameters | AIC | dAIC |
| A | -163743.43 | 17 | 327520.864 | 99.6025137 |
| B | -163787.96 | 17 | 327609.919 | 188.657295 |
| C | -163708.42 | 19 | 327454.838 | 33.5762958 |
| D* | -163691.63 | 19 | 327421.262 | 0 |
|  |  |  |  |  |
| 6-Pop |  |  |  |  |
| Model | ln lhood | N parameters | AIC | dAIC |
| A | -158727 | 21 | 317495.999 | 114.219324 |
| B | -158902.38 | 21 | 317846.752 | 464.972111 |
| C* | -158667.89 | 23 | 317381.78 | 0 |

Table S3 Inferred parameters (point estimate and confidence intervals calculated with parametric bootstrapping) of the most likely model for the 6-POP dataset. Ne – effective population size of the Alpine population (ALP), Baltic (BAL), Norwegian (NOR), Scottish from Lomond (LOM), Wales (LTE), English from Red Tarn (RTA), and the ancestral population (ANCSIZE). PRLW = admixture proportion from Scottish to Welsh, PRWB = admixture proportion from Wales to Baltic, PRLB = admixture proportion from Scottish to Baltic, PRBL = admixture proportion from Baltic to Scottish, PRAB = admixture proportion from Alpine to Baltic, PRNB = admixture proportion from Norwegian to Baltic. TD is divergence time in generations; TD4 = divergence time of the Norwegian population, TD3 = divergence time of the Baltic population, TD2 = divergence time of the Lomond population, TD1 = divergence time of the Welsh population, TD0 = divergence time of the English population. TA - time of admixture, in generations, between Norwegian and Baltic populations (NB), Alpine and Baltic (AB), Baltic and Scottish (BL), Welsh and Baltic (WB), Scottish and Welsh (LW).

| Parameter | Point estimate | Confidence intervals |
| --- | --- | --- |
| ALP (Ne) | 10246 | 10246 - 11585 |
| BAL (Ne) | 104380 | 98887 - 135117 |
| NOR (Ne) | 9367 | 9226 - 10372 |
| LOM (Ne) | 7267 | 6783 - 8063 |
| LTE (Ne) | 11659 | 9879 - 11659 |
| RTA (Ne) | 7212 | 7030 - 8153 |
| ANCSIZE (Ne) | 74987 | 71904 - 74987 |
| PRLW | 0.1515633 | 0.147 – 0.152 |
| PRWB | 0.1772821 | 0.172 – 0.177 |
| PRLB | 0.1503929 | 0.150 – 0.150 |
| PRBL | 0.0880333 | 0.088 – 0.088 |
| PRAB | 0.3725121 | 0.373 – 0.373 |
| PRNB | 0.4597753 | 0.460 – 0.460 |
| TD4 | 26247 | 23451 - 26774 |
| TD3 | 23205 | 20733 - 23671 |
| TD2 | 18674 | 16685 - 19435 |
| TD1 | 5965 | 5313 - 6255 |
| TD0 | 3887 | 3337 - 4259 |
| TANB | 6453 | 5765 - 6582 |
| TAAB | 6211 | 5549 - 6336 |
| TABL | 6663 | 5953 - 6934 |
| TAWB | 4433 | 3949 - 4649 |
| TALW | 3473 | 3093 - 3641 |
